# Immunomodulation of Antibody Glycosylation through the Placental Transfer

**DOI:** 10.3390/ijms242316772

**Published:** 2023-11-26

**Authors:** Chang Gao, Qingyan Chen, Xinxin Hao, Qiushi Wang

**Affiliations:** Department of Blood Transfusion, Shengjing Hospital of China Medical University, Shenyang 110004, China

**Keywords:** antibodies, immunoglobulin G, glycosylation, placental transfer, Fc receptor (FcR), pregnancy

## Abstract

Establishing an immune balance between the mother and fetus during gestation is crucial, with the placenta acting as the epicenter of immune tolerance. The placental transfer of antibodies, mainly immunoglobulin G (IgG), is critical in protecting the developing fetus from infections. This review looks at how immunomodulation of antibody glycosylation occurs during placental transfer and how it affects fetal health. The passage of maternal IgG antibodies through the placental layers, including the syncytiotrophoblast, stroma, and fetal endothelium, is discussed. The effect of IgG subclass, glycosylation, concentration, maternal infections, and antigen specificity on antibody transfer efficiency is investigated. FcRn-mediated IgG transport, influenced by pH-dependent binding, is essential for placental transfer. Additionally, this review delves into the impact of glycosylation patterns on antibody functionality, considering both protective and pathological effects. Factors affecting the transfer of protective antibodies, such as maternal vaccination, are discussed along with reducing harmful antibodies. This in-depth examination of placental antibody transfer and glycosylation provides insights into improving neonatal immunity and mitigating the effects of maternal autoimmune and alloimmune conditions.

## 1. Introduction

In pregnancy, the placenta serves as the key to immune tolerance and housing the primary protective mechanisms against fetal rejection [1,2]. The histological barrier placenta provides essential nutrients while facilitating waste transport and promoting fetal growth and development. Multiple active and passive mechanisms allow substances, such as oxygen, glucose, amino acids, and other nutrients, to be exchanged [3,4]. Immunoglobulin G (IgG) is the only immunoglobulin class that crosses the placenta. While the fetus can produce a small amount of IgG, maternal transport via the placenta is the primary source of IgG in umbilical cord blood [5,6]. The placenta is a complex organ with the chorionic villus as its basic unit [4]. Maternal IgG antibodies must travel through three distinct layers of the placenta to reach the fetal circulatory system. The syncytiotrophoblast, the intervillous stroma containing placental fibroblasts and Hofbauer cells, and the fetal vascular endothelium are all traversed during this journey. Among these layers, antibody transfer across the syncytiotrophoblast is significant in ensuring immune protection for the developing fetus [7,8,9].

**Figure 1:**
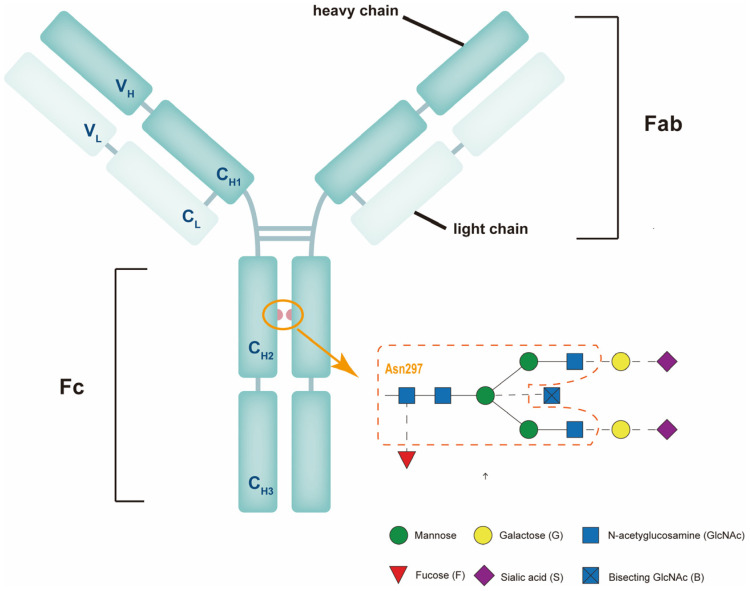
A schematic illustration of immunoglobulin G (IgG) and the Fc glycan modification. IgG antibodies comprise two heavy and two light chains, forming antigen-binding (Fab) and effector function (Fc) domains. The Fc domain has an N-glycosylation site at asparagine 297 (Asn297), highlighted with an orange circle. The orange arrow in the diagram points to the detailed glycans. The glycan attached at Asn297 has a core heptasaccharide with four N-acetylglucosamine (GlcNAc, blue squares) and three mannose (green circles) residues, indicated by an orange dashed line, and may contain additional glycans such as fucose (red triangle), bisecting GlcNAc (blue square), galactose (yellow circles), and sialic acid (purple diamonds).

Immunoglobulins are the predominant glycoproteins in the immune system, with IgG being the most abundant in serum. The IgG molecule is a Y-shaped structure comprising four polypeptide chains, two identical 50 kDa heavy (H) chains, and two identical 25 kDa light (L) chains. Each chain is composed of both amino-terminal variable (V) and constant (C) domains. Each heavy chain contains one variable domain (VH) and three constant domains (CH1, CH2, and CH3). Similarly, the light chain contains a variable domain (VL) and a constant domain (CL). The antigen-binding parts are known as Fab fragments, which comprise the VL and CL light chain and the VH and CH1 heavy chain domains [10]. The Fc fragment is the remaining IgG molecule portion corresponding to the CH2 and CH3 heavy chain domains (Figure 1). Fc fragments do not recognize pathogens; instead, they mediate effector function and molecular interactions, such as antibody-dependent cytotoxicity and immune phagocytosis [11]. The hinge region is the amino acid sequence that connects the Fab and Fc fragments, allowing the antibody to be flexible [12].

Because of their vulnerability, neonates are still at risk of infection and even death. IgG antibodies, known for their critical functions, protect the infant against a wide range of pathogens by improving the functionality of immune cells and thus fortifying the developing infant’s immune defense mechanisms [13]. The half-life of maternal antibodies determines how long they are present in the infant at protective levels [14]. The process of placental transfer begins during the early stages of pregnancy, usually around the 13th week of gestation. Fetal IgG levels rise gradually as gestational age increases. Fetal IgG concentrations remain relatively low during the mid-trimester period, roughly within 16 weeks, accounting for approximately 10% of maternal levels. However, by the time the pregnancy reaches term, fetal IgG levels have surpassed maternal plasma IgG levels at birth [14]. Preterm infants receive less transplacental transfer of maternal IgG, such as that targeting the varicella-zoster virus, than full-term infants, according to studies on maternal vaccinations. It causes a faster decline in IgG concentrations in preterm infants, causing them to reach non-protective levels sooner and making them more vulnerable to vaccine-protected diseases at a younger age. While the half-life of maternal varicella-zoster virus IgG in full-term infants is 42 to 45 days, it is much shorter in preterm infants, standing at only 25.5 days [15], and antibodies transferred from the mother can enhance the fetal immune response, amplifying its innate capability to produce protective antibodies [16].

Several factors influence the efficiency of IgG transfer during pregnancy, including IgG subclass, glycosylation, concentration, maternal infections, and antigen specificity. Placental integrity also plays a vital role in mediating this transfer.

IgG antibodies serve as vital bridges between the host immune response and antigens. The effectiveness of IgG antibodies depends on several factors, most notably their subclass and Fc glycosylation, which significantly impact functional activity. Glycosylation has been linked to anti-inflammatory responses and effector function activities, such as antibody-dependent cellular cytotoxicity (ADCC) [17]. When IgG antibodies cross the placenta, these intricate considerations come into play. However, the transferred antibodies’ effector functions play a dual role. On the one hand, they are poised to facilitate microbe clearance, thereby protecting the fetus. These effector functions, on the other hand, may inadvertently target self-antigens or antigens of paternal origin, potentially leading to immune responses against the host’s tissues. This review summarizes glycosylated antibody passage mechanisms and functional activities through the placenta.

## 2. Physiological Transport of IgG across the Placenta

The neonatal Fc receptor (FcRn) is a critical mediator of IgG transport across the placental barrier, which is required for maintaining IgG homeostasis during pregnancy. This method is based on a pH-dependent mechanism. During pregnancy, the fetus receives maternal IgG via a process known as transcytosis. The interaction of the FcRn with IgG is highly pH-sensitive, with high affinity at acidic pH levels (below 6.5) and low affinity at physiological pH levels (around 7.4) [18]. Syncytiotrophoblast cells, which are found in the placenta, are essential in this process. Through fluid-phase endocytosis on the apical side, they actively take maternal IgG from the extracellular fluid. In an acidic environment, this internalization results in the formation of endosomes. IgG binds to the FcRn within these endosomes, preventing its degradation by lysosomal enzymes [19,20,21]. Endosomes containing IgG–FcRn complexes are then delivered to the basal cell membrane. In this case, exposure to the neutral pH of the stroma causes FcRns to release IgG. This critical step allows IgG to enter the fetal circulation and thus reach the developing fetus [22,23]. The FcRn is structurally like a molecule related to the major histocompatibility complex class I (MHC) [24,25]. It binds IgG at the CH2–CH3 interface of the constant region without causing the antibody to change conformation. The FcRn exists as a dimer, with high-affinity binding at acidic pH levels, which is due in part to histidine-mediated salt bridges and a sidechain rearrangement that improves interactions with anionic pockets [20]. The FcRn’s pH-dependent binding of IgG is critical to its role in IgG transport. It protects IgG from degradation in the acidic endosomal environment and ensures that it is released at the appropriate site in the placental stroma. Understanding the complexities of this pH-dependent mechanism is crucial for elucidating the physiology of maternal–fetal IgG transfer and has clinical implications. It may be possible to improve the pharmacokinetics of therapeutic antibodies or reduce harmful antibodies in immune conditions by modulating the IgG–FcRn interaction, ultimately benefiting clinical practice and patient outcomes.

**Figure 2:**
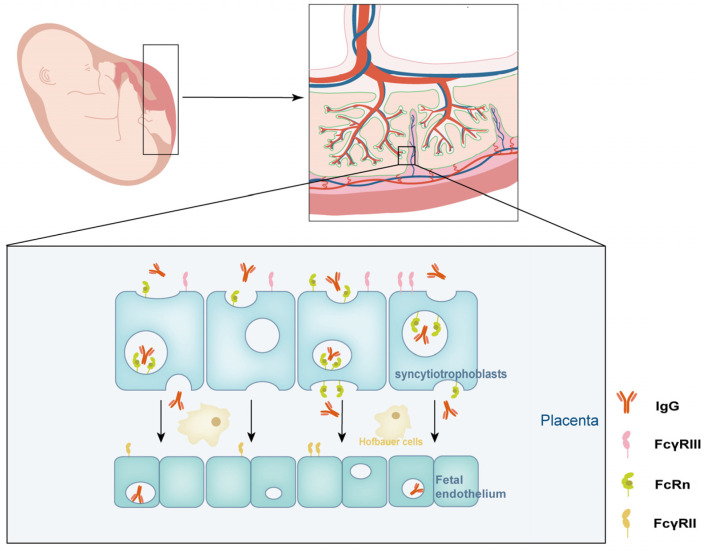
The IgG transfer process and outcomes from mother to fetus. According to the diagram, the neonatal Fc receptor (FcRn) promotes active transport of maternal IgG across the chorionic villus zones, mainly through syncytium. While the precise mechanisms of IgG transfer through the placental stroma and fetal endothelium are unknown, they are most likely influenced by other Fcγ receptors found in Hofbauer and endothelial cells.

However, the precise mechanisms that allow IgG to cross the villous stroma and the fetal vessel endothelium remain unknown. Hofbauer cells, fibroblasts, and connective tissue are found within the stroma, with Hofbauer cells acting as fetal macrophages and interacting with IgG via various Fc receptors. IgG is found in abundance in both Hofbauer cells and other stroma. Stromal macrophages with FcγRI, FcγRII, and FcγRIII receptors can bind immune complexes but not prevent monomeric IgG from reaching fetal capillaries. This indicates that not all maternal IgG crosses the placenta effectively [5,24]. Human placental endothelium serves as an essential protective barrier. IgG crosses the fetal vessel endothelium via a transcellular pathway rather than a paracellular route. This mechanism makes sure that only certain substances reach the developing fetus. Endothelial cells can bind to immune complexes, preventing them from reaching the fetus. It is unclear whether the FcRn is expressed in fetal vessel endothelium, but evidence suggests that alternative Fc receptors are involved in this process [24]. The FcγRII receptor is prominently in the terminal villous vessel endothelium at term. The subtype, FcγRIIb, does not bind well to individual IgG molecules but rather interacts with immune complexes, implying that it plays a role in preventing immune complexes from entering the fetal circulation. The fetal endothelium contains unique vesicles rich in FcγRIIb and IgG, which are impermeable to trans-junctional movements. Even in the absence of the FcRn on the fetal endothelium, IgG passes through, implying the presence of other facilitating receptors or methods. IgG-carrying vesicular structures on the cell surface highlight a complex IgG transport mechanism across this barrier (Figure 2) [24,26].

The maternal immune system undergoes profound changes during pregnancy to protect and support the developing fetus, which is semi-allogeneic. The clonal selection of IgG antibodies is a critical adjustment. While IgG is essential in protecting against pathogens, its importance is magnified during pregnancy. It accomplishes this by altering the diversity and function of its T and B lymphocyte populations. In this situation, it acts as a shield, preventing the mother’s immune system from rejecting fetal elements. Antibodies that may harm the fetus are reduced during the clonal selection process, while those critical for defending the fetus against potential infections are increased. The goal is simple: reduce the production of antibodies that could harm the fetus [27]. Furthermore, due to glycosylation at one of its Fab arms, the placenta can produce a specific type of IgG known as asymmetric IgG (aIgG). This specialized IgG form can interact with a variety of antibodies as well as specific leukocytes. Such interactions are critical in modulating local immune responses at the interface between two genetically distinct entities, ensuring harmonious coexistence and protection [28]. It creates a long-term tolerance for specific idiotypes. The number of selected clones of IgG that were beneficial during pregnancy gradually decreases after pregnancy because they are no longer required at the same levels. Maternal IgG transferred to the fetus during pregnancy, on the other hand, protects the newborn during the first few months of life until the infant’s immune system produces its antibodies.

## 3. Factors Influencing Transplacental Antibody Transfer

### 3.1. IgG Subclass

IgG subclasses are classified based on differences in the constant region of the heavy chain: IgG1, IgG2, IgG3, and IgG4 [29]. IgG1 is the most common subclass, accounting for 60% of serum IgG; IgG2 makes up 32%; and IgG3 and IgG4 constitute 4% [30]. The length and rigidity of the hinge region differ significantly between subclasses, and their relative flexibility varies as follows: IgG3 > IgG1 > IgG4 > IgG2 [31]. The concentration and flexibility of IgG subclasses influence their placental transfer. The maternal IgG subclass transfer efficiency reveals that IgG1 is preferentially transferred, followed by IgG4, IgG3 (low abundance), and IgG2 (low transfer) [7,19,32]. However, the transport profile of G3 allotypes, such as G3m16+ individuals expressing H435-containing IgG3 [33], closely resembles that of IgG1. This distinct transport behavior may lead to increased complications in alloimmune diseases associated with pregnancy [34].

### 3.2. IgG Glycosylation

In IgG molecules, each Fc domain contains a glycosylation site at asparagine 297 (Asn297), which maintains structural stability and regulates protein spatial conformation [35,36,37,38]. In addition to the conserved glycosylation site Asn297 in the CH2 domain found in all IgG subclasses, IgG3 has an additional N-glycosylation site in the CH3 domain at Asn392 [39]. The core structure of IgG glycan is a heptasaccharide composed of four N-acetylglucosamine (GlcNAc) and three mannose residues that can be extended with galactose, sialic acid, core fucose, and bisected GlcNAc (Figure 1) [40,41]. Each H receives a high-mannose glycan during the antibody synthesis process within the endoplasmic reticulum. The glycans are meticulously modified as this antibody advances to the Golgi apparatus. Glc3Man9GlcNAc2, the precursor glycan, first binds to the antibody polypeptide in the endoplasmic reticulum. When a glycosylated molecule reaches the Golgi, it is met by a carefully orchestrated sequence of glycosidases and glycosyltransferases. These enzymes sequentially trim and augment the glycan, resulting in the antibody’s signature biantennary structure. To begin this modification, the glycan is reduced to a Glc4Man3 precursor in the cis and medial Golgi sections. Subsequently, several glycosyltransferases are activated: FUT8 adds fucose to the medial Golgi; B4GALT1 appends up to two galactoses in the trans-Golgi; MGAT3 incorporates bisected GlcNAc; andST6GAL1 incorporates one or two sialic acids. The complexity of antibody glycosylation is exemplified by this intricate process [42]. During a healthy pregnancy, the level of galactosylation and sialylation in the IgG Fc region increases, with a slight decrease in the fucosylated IgG Fc portion [43,44,45]. Several early studies have shown a decrease in the concentration of non-glycosylated IgG and an increase in the concentration of galactosylated IgG in neonates, indicating that glycosylated antibodies are preferentially transported (Table 1) [46,47]. The FcRn has been shown to transport IgG precisely [48]. FcRn chromatography has revealed that glycosylation patterns can influence FcRn interactions, with deglycosylated IgG exhibiting lower binding [49]. Other research has found that the interaction between IgG and FcRn is not dependent on glycosylation, and thus, IgG transport does not favor Fc glycosylation [50,51,52]. Furthermore, Fc galactosylation does not affect FcγR- or FcRn-binding in IgG subclasses [53]. Disagreements in the findings of various studies may be related to the IgG subclass, antigen specificity, placental receptor expression, and experimental methods. Mice and rats are the most used models for studying placental transfer. Unlike the human placenta, which serves as the primary pathway of passive immunity, mice and rats transfer IgG primarily through postnatal breast milk [25,54,55]. This distinction may limit the use of mouse and rat models in human research.

### 3.3. FcR

FcR is a cell surface protein that binds specifically to an antibody’s Fc fragment. The three types of FcRs, known as FcγRI, FcγRII, and FcγRIII, are IgG receptors based on their affinity for Fc regions. FcγRI is expressed on monocytes/macrophages and dendritic cells (DC) and is involved in neutrophils and mast cell activation [56]. FcγRIIa is expressed by all myeloid cells. FcγRIIb is abundantly expressed in basophils and circulating B cells [57]. FcγRIIc is expressed in natural killer (NK) cells, monocytes, and neutrophils [58]. FcγRIIIa is expressed in NK cells and monocytes/macrophages. FcγRIIIb is found primarily in neutrophils [59]. FcRn is expressed by antigen-presenting cells, monocytes/macrophages, neutrophils, vascular endothelial cells, intestinal epithelial cells, and, most importantly, syncytiotrophoblasts [20,60]. Trophoblasts contain significant FcγRIII (40.70%) and FcRn (22.40%). FcRn is almost not expressed by Hofbauer cells or fetal epithelial cells [61]. Based on the signaling properties of their intracellular portions, FcγRs can be classified as either activating or inhibitory. The presence of immunoreceptor tyrosine activating motifs (ITAMs) distinguishes activating receptors, which include FcγRI, FcγRIIa, FcγRIIc, and FcγRIIIa. On the other hand, FcγRIIb is the only inhibitory FcγR distinguished by an immunoreceptor tyrosine inhibitory motif (ITIM) located in its intracellular domain. FcγRIIIb is unique because it is a GPI-anchored protein with no direct signaling capabilities. Despite this, it can still facilitate activation signals, notably when crosslinked, and it frequently collaborates with activating receptors, most notably FcγRIIa [62]. FcRn has been identified as the primary mechanism underlying IgG transplacental transport. Other FcγRs may also play a role in this process. One critical function of FcγRs is to act as protective barriers, ensuring that immune complexes do not reach the developing fetus, highlighting the critical role of FcγRs in fetal health.

### 3.4. IgG Concentration

The concentration of IgG has a significant impact on placental transfer, resulting in a paradoxical interplay. Maternal antibody levels are essential determinants of transfer efficiency, with neonatal IgG levels typically mirroring maternal ones. However, when maternal total IgG levels exceed a predefined threshold (>15 g/L), as is common in hypergammaglobulinemia, this leads to FcRn oversaturation, resulting in the degradation of unbound IgG molecules and, ultimately, a reduction in fetal antibody transfer [19,63]. IgG must compete for a limited number of FcRns in this scenario. Several studies in African populations have found that lower IgG transfer ratios are associated with higher maternal total IgG levels [64,65]. Cord sera typically had lower total IgG concentrations than maternal sera when the total IgG levels of maternal sera reached 15 g/L. This is consistent with previous studies that found significant negative correlations between maternal IgG levels and placental transfer ratios to neonates for total IgG and specific IgG antibodies targeting different antigens [66,67].

### 3.5. Maternal Immune Response

There has been a decrease in the transfer of maternal–fetal antibodies in pregnant women with both acute and chronic infectious diseases, such as human immunodeficiency virus (HIV), malaria, and severe acute respiratory syndrome coronavirus 2 (SARS-CoV-2) infection [68,69,70,71,72,73,74]. Several mechanisms have been proposed to explain decreased transport in maternal coinfections. These include hypergammaglobulinemia, which may saturate placental FcRns, changes in glycan structures, and inflammation-induced changes in the placenta. Notably, when compared to healthy pregnant individuals, the glycosylation patterns of IgG play a significant role in reducing the efficiency of placental transfer in HIV-infected pregnant women. Women with HIV had different antibody profiles during pregnancy than women who tested negative for HIV. Pregnant women with HIV had elevated levels of agalactosylated antibodies. The presence of digalactosylated and sialylated antibodies, on the other hand, was reduced (Table 1) [75]. The decreased galactosylation seen in the antibodies of HIV-positive pregnant women may be directly related to the decreased efficiency of antibody transfer. The compromised maternal–fetal antibody transfer in cases of infection during pregnancy may have implications for neonatal immunity and susceptibility to infections. This highlights the significance of understanding the impact of infectious diseases on the dynamics of IgG transfer across the placenta, as well as the specific glycosylation patterns that can influence these processes.

## 4. Placenta Transfer of Protective IgG

### 4.1. Vaccination during Pregnancy

The administration of vaccines for newborn immunization is critical. Currently, two vaccine strategies are available to protect infants: the first is to vaccinate infants and others who may be exposed to sources of infection, and the second is to vaccinate pregnant women, which provides direct protection through the passive transfer of antibodies [76,77,78,79]. While increased antibody transfer improves fetal and neonatal immunity, decreased maternal antibodies limit the protection of neonatal illness [80,81]. It has been demonstrated that NK cell-activating antibodies preferentially transfer to neonates during pertussis immunization. Other virus-specific antibodies, such as those against respiratory syncytial virus (RSV), influenza (Flu), and measles, are also transferred preferentially [61]. Fetal antibody concentrations are affected by gestational age, maternal antibody levels, antigen specificity, IgG subtypes, and IgG glycosylation [82,83]. Fetuses typically receive maternal antibodies during the first trimester. These antibodies in the newborn begin to decline after childbirth. Maternal antibody levels for RSV typically decline between 2 and 6 months. While influenza can last a few months, there is a significant reduction around 4–6 months after birth. Antibodies from the mother against measles generally decrease over 6–12 months. Antibodies from the mother specific to pertussis typically begin to decline around the 6-month mark. Pertactin (PTN), filamentous hemagglutinin (FHA), fimbriae (FIM), and pertussis toxin (PTX) are the four antigens in the pertussis vaccine [84]. The preference for IgG1 transfer in IgG is consistent with previously described IgG subclass transfer. However, the FIM-specific IgG1 transfer ratio is lower, indicating that factors other than antibody subclass influence placental transfer. Agalactosylated and bisected antibodies are systematically inhibited, whereas galactosylated and sialylated antibodies significantly increase antigen-specific proteins (Table 1). Although Fc glycosylation does not affect FcRn-binding, antibodies with digalactosylation bind more potently to FcRn and FcγRIIIa in pertussis-specific immunization, where FcγRIII may have a synergistic effect on IgG binding to FcRn [61]. Therefore, the mechanism of preferential transfer of antibodies involved in NK cell activation for pertussis-specific immunity is based on the modification of IgG antibody glycosylation, where double-galactosylated antibodies bind efficiently to FcRn and FcγRIIIa, facilitating the induction of NK cell function in the fetus after placental transfer.

Antibody changes in response to influenza vaccination during pregnancy are similar. Pregnant women’s total antibody Fc glycan profile, which includes increased fucosylation and sialylation as well as enhanced digalactosylated hemagglutinin (HA)-specific antibodies, is consistent with reduced inflammation, maintenance of antiviral effector functions, enhanced placental transfer, and newborn protection [85].

### 4.2. Maternal Infection

During the pandemic, pregnant women were more likely to be infected with SARS-CoV-2, though definitive proof of vertical transmission and placental infection is lacking [74,86]. Many studies have shown that infants can effectively receive the antibodies that the mother produces following SARS-CoV-2 infection during pregnancy [87,88,89]. Total IgG transfer levels in infected pregnant women were no different from those in uninfected pregnant women in a study of placental antibody transfer. Non-SARS-CoV-2 placental antibody transmission is normal in the presence of a maternal SARS-CoV-2 infection, but the placental transfer of SARS-CoV-2-specific antibodies is reduced [74]. Glycan profiles in the IgG Fc region of SARS-CoV-2-specific antibodies have been linked to impaired SARS-CoV-2-specific antibody transfer in pregnant women infected with the virus [90]. A comparison of differences in total IgG and SARS-CoV-2-specific IgG antibody glycosylation in infected pregnant women revealed that SARS-CoV-2-specific antibodies had an increased proportion of digalactosylation and fucosylation and a decreased proportion of agalactosylation and bisected GlcNAc antibodies. As previously described, IgG antibodies with high galactosylation and low fucosylation levels show a trend toward preferential placental transfer. The placental transfer of digalactosylated SARS-CoV-2-specific antibodies is preferential at similar rates, which are above one, as the placental transfer of total IgG. In contrast, the higher proportion of fucosylated SARS-CoV-2-specific antibodies compared to total IgG, as well as the higher proportion of fucosylated antibodies compared to digalactosylated antibodies, may explain the reduced placental transfer of SARS-CoV-2-specific antibodies. However, in infected and non-infected pregnant women, the transfer ratio of agalactosylated, sialylated, and bisected GlcNAc SARS-CoV-2-specific antibodies increases, with agalactosylated/afucosylated (G0) antibodies selectively transferred but agalactosylated/fucosylated (G0F) antibodies retained in the mother [49]. This indicates that decreased SARS-CoV-2-specific antibody transfer is due to an inclination toward the glycoforms found predominantly on SARS-CoV-2-specific antibodies [90]. ‘Antibody glycoforms’ are antibody variations caused by different carbohydrate structures, or glycans, attached to them. In particular, the Fc region of IgG antibodies contains a glycosylation site where these glycans attach. The composition of these attached carbohydrates is critical because it significantly impacts antibody functions. Different glycoforms are formed due to variations in attached glycans, influencing how the antibody interacts with other cells and its overall efficacy, resulting in various effects on the immune response. This antibody transfer characteristic may be related to maternal protective immunity against SARS-CoV-2 to retain massive amounts of antibodies in the mother to protect against viral attack. Thus, antibodies may protect maternal immunity in infectious diseases by altering glycosylation levels and reducing placental transfer to retain maternal pathogen antigen-specific antibodies.

The occurrence of neonatal herpes simplex virus (HSV) infection has recently increased. The emergence of HSV as the leading cause of primary genital infections is a significant factor driving this rise [91]. When primary maternal infection occurs during the third trimester, the antibody profile produced exhibits a variety of glycosylation patterns. Anti-glycoprotein B (gB) antibodies with neutralizing activity contain more terminal Fc-sialylated glycans. On the other hand, ADCC-mediating anti-gB antibodies with concurrent SARS-CoV-2 infection are characterized by increased fucosylated, bisecting GlcNAs, and digalactosylated glycans. Antibody glycosylation patterns in antibodies point towards distinct affinities for their corresponding receptors. An increased colocalization of FcRn and FcγRIIIa within the placenta supports this association. Given that only minimal levels of ADCC-mediating antibodies are produced in response to primary HSV infection, this inefficient transfer may contribute to an increase in neonatal disease risk, particularly in cases of primary maternal infection. Monoclonal antibodies should be engineered to contain glycans that simultaneously enhance FcRn-binding and FcγRIII-activation for optimal placental transfer, emphasizing the importance of vaccines prioritizing monoclonal antibodies with ADCC-mediating activity targeting gB [92].

Research shows that HIV-infected women have less efficient placental transfer of protective IgG. Inefficiently transferred gp120-specific IgG has an elevated presence of fucose in the Fc region. This suggests that Fc region glycans play a role in influencing placental IgG transfer efficiency, possibly through changes in binding to placental Fc receptors like FcγRIIIa. Consequently, HIV-exposed but uninfected infants of these mothers may be more vulnerable to diseases that can be avoided with vaccinations [93].

## 5. Placenta Transfer of Pathological IgG

Antibodies, in addition to neutralizing pathogens, stimulate a variety of immune responses, including antibody-dependent cellular phagocytosis (ADCP), ADCC, and complement-dependent cytotoxicity (CDC) [42,94]. These effector functions are influenced by antibody glycosylation, which affects their ability to bind Fc receptors on various immune cells. The findings show that changing the glycosylation patterns of IgG1 impacts its binding to FcγRIIIa significantly. Reduced fucosylation enhances its binding affinity by about 17 times. This increase can be amplified nearly 40 times with increased galactosylation, augmenting NK cell-driven antibody-dependent cellular cytotoxicity. It is worth noting that NK cells are the only cells that express FcγRIIIa exclusively. Increased affinity for FcγRIIIa also leads to amplified ADCC activity in mononuclear cells [30]. Furthermore, regardless of the fucosylation status, increased galactosylation and sialylation significantly enhanced C1q-binding, triggering subsequent complement deposition and cytotoxic effects. These differences in binding affinities between FcγRIIIa and IgG1 are consistently reflected in differences in cell-mediated functions and their corresponding in vivo activities [95,96].

### 5.1. Alloimmune Diseases

Antibody-mediated effector interactions, such as ADCC and ADCP, bind to antigens on normal fetal cells, causing cytotoxicity or phagocytosis, which can result in maternal–fetal alloimmune disorders such as hemolytic disease of the fetus and newborn (HDFN) and fetal or neonatal alloimmune thrombocytopenia (FNAIT), both of which are caused by blood group incompatibility. Some studies have found that antibody glycoforms are related to disease severity. Einarsdottir et al. concluded, in a study of HDFN and FNAIT, that fetal and maternal IgG have similar glycosylation levels [50] and that IgG Fc-glycans are not involved in FcRn-binding [53]. Hence, glycosylation of IgG does not affect this process. In contrast, Jennewein et al. show that placental sieving is required for antibody transfer via altered glycosylation, and antibodies transferred through the placenta continue to interact with target cells [61].

#### 5.1.1. HDFN

Human red blood cells express a multitude of blood group antigens, including the ABO, Rh, and Kell system. HDFN is a complicated condition in which maternal Red Blood Cell (RBC)-specific alloantibodies may destroy fetal erythrocytes, resulting in neonatal anemia and hyperbilirubinemia. In severe cases, it can cause conditions such as hydrops, kernicterus, and even death [97]. Several factors influence the severity of HDFN, primarily alloantibody characteristics such as concentration, subclass, glycosylation modification, and RBC antigen [98]. The Rh system includes antigens, including D, E, e, C, and c, with D being fully developed at birth and exhibiting the highest immunogenicity [99]. The predominant glycoforms in maternal anti-D-specific IgG1 during pregnancy are decreased core fucosylation, increased galactosylation, and reduced bisection [100]. In the Kell system, severe fetal anemia, like anti-D, is associated with decreased Fc-fucosylation of anti-K antibodies. However, the severity of HDFN disease is associated with increased Fc-galactosylation and sialylation in anti-c antibodies [99]. Depending on the antigen, the glycosylation pattern of IgG-Fc on anti-red blood cell antibodies varies. Many studies have shown that decreasing fucosylation enhances the affinity with which antibodies bind to receptors such as FcγRIII, thereby augmenting the efficacy of ADCC [101,102,103,104]. As previously stated, antibodies with high galactosylation are more easily transported, activating NK cells (Table 1). Pregnant women with elevated antibody levels, decreased fucosylation, and increased galactosylation are at risk of developing HDFN.

#### 5.1.2. FNAIT

FNAIT is a hemorrhagic disorder in which maternal platelet-specific antibodies cross the placenta and bind to fetal or neonatal platelets expressing the corresponding antigens, resulting in platelet immune destruction [105,106]. Most antibodies are anti- human platelet antigen (HPA)-1a, mostly IgG1 and IgG3; however, there is no evidence that this affects the severity of FNAIT [107,108]. Anti-HPA-1a antibody glycosylation levels were discovered to be promising diagnostic markers by Kapur et al. [109]. FNAIT, like HDFN, showed decreased core fucosylated anti-HPA-1a IgG1-Fc levels as well as elevated galactosylation and sialylation [110,111], and anti-HPA-1a IgG1 fucosylation was negatively correlated with clinical severity of FNAIT, with all asymptomatic patients exhibiting high levels of anti-HPA-1a Fc fucosylation [109]. Unlike the stimulatory effect of reduced anti-HPA-1a fucosylation, highly galactosylated and sialylated anti-HPA-1a antibodies may suppress the immune response (Table 1).

### 5.2. Autoimmune Diseases

During pregnancy, autoimmune diseases caused by maternal antibodies show varying degrees of inflammatory activity, which can affect the developing fetus. The modifications of IgG antibodies intricately influence these antibody-induced inflammations. IgG glycosylation, a key factor in many biological processes, is linked to autoimmune diseases like systemic lupus erythematosus (SLE) and antiphospholipid antibody syndrome (APS). It is noteworthy that non-galactosylated (G0) and non-sialylated IgG antibodies are associated with pro-inflammatory conditions in autoimmune diseases [112]. Conversely, the presence of galactose and sialic acid attachments on IgG antibodies is associated with anti-inflammatory effects. SLE research has revealed abnormal IgG glycosylation patterns during pregnancy. Specifically, SLE patients have significantly lower sialylation levels in their IgG antibodies during this time. This abnormally low sialylation level is linked to two critical symptoms: SLE activity and the risk of fetal death during pregnancy. Further research has revealed that reduced sialylation impairs the ability of IgG to inhibit DC function, which is critical in autoimmune diseases [113]. A comprehensive glycosylation analysis was performed on purified anti-β2GPI IgG and total IgG in the context of APS research. The findings highlighted the close relationship between the anti-β2GPI IgG glycosylation profile and clinical manifestations of APS. Anti-β2GPI IgG exhibited lower levels of galactosylation, higher levels of bisecting structures, and core fucosylation, all of which have been linked to catastrophic antiphospholipid antibody syndrome (CAPS) and triple-positive antiphospholipid antibodies (aPLs) [114]. These findings emphasize the critical role of IgG glycosylation in autoimmune diseases during pregnancy. Anomalies in sialylation are associated with SLE activity, whereas glycosylation modifications such as galactosylation are closely linked to the clinical features of APS (Table 1). These findings contribute to a better understanding of the pathological mechanisms underlying autoimmune diseases during pregnancy, as well as provide new fresh avenues for future research into diagnosis and treatment.

Passive immunity is provided to the fetus by transporting IgG antibodies across the placenta. When pathological IgG is present, however, it can cause maternal–fetal complications. Antibody glycosylation is essential in this process. For example, changes in fucosylation and galactosylation affect how antibodies bind to Fc receptors on immune cells. Immune responses, such as ADCC, can be enhanced or diminished by altered binding. Thus, pathological antibodies can cause diseases, and the glycosylation pattern of antibodies may influence the severity of these diseases. The delicate balance of IgG glycosylation modification plays a critical role in antibody transport across the placenta and the potential implications for fetal health.

## 6. Antibody Therapy and Future Perspective

There are two promising applications for studying the mechanisms of antibody transfer in the placenta. One strategy involves enhancing the efficiency with which protective antibodies are transferred. Maternal vaccination is a novel approach to boosting neonatal immunity. Understanding the mechanism of placental antibody transfer is critical for developing maternal vaccines aimed at enhancing neonatal immunity. There is much evidence that antibody glycosylation could potentially regulate antigen specificity. Stimulation by specific antigens can modulate antibody glycosylation, enhancing the protective role of vaccines. While influenza and tetanus vaccines do not affect total IgG glycosylation, antigen-specific IgG shows increased levels of galactosylation and sialylation after active immunization. This implies that B lymphocytes can produce IgG with distinct Fc N-glycosylation patterns [115]. When HIV vaccines are administered to populations in different geographic regions, significant variations in IgG glycosylation are observed among individuals. Conversely, immunization can overcome these differences and induce antigen-specific antibodies with similar glycosylation profiles. Furthermore, different vaccine regimens result in distinct antigen-specific IgG glycosylation patterns, indicating that antibody glycosylation is programmable and can be manipulated during B cell priming by transmitting different inflammatory signals [116]. Furthermore, glycosylation regulation can be orchestrated through endogenous pathways, such as sialylated anti-HA antibodies activating B cell receptors (BCR), promoting the production of high-affinity neutralizing antibodies [117].

The other use is to reduce harmful antibodies; antibody glycosylation has been linked to the severity of clinical manifestations of both alloimmune and autoimmune disorders. In RhD HDFN immunization, anti-RhD antibodies enhance the NK cell activation, thereby promoting immune suppression by eliminating DCs and, as a result, preventing B cells from producing anti-RhD antibodies. Removed glycosylation from anti-RhD antibodies, such as core fucosylation, abolishes their ability to induce NK cell degranulation, indicating that anti-RhD antibodies activate NK cells via glycosylation-dependent Fc segments [118]. Furthermore, research suggests that adding sialylation to intravenous immunoglobulin for intravenous injection improves stability and anti-inflammatory activity [119].

## 7. Conclusions

The placental transfer of antibodies, mainly IgG, is a complex and vital process that protects the developing fetus from infections. This review examined the mechanisms and factors influencing antibody glycosylation immunomodulation during placental transfer. We discussed how antibodies, primarily IgG, travel through the placental layers to reach the fetal circulatory system, including the syncytiotrophoblast, stroma, and fetal endothelium. The efficiency of this transfer is influenced by factors such as IgG subclass, glycosylation patterns, concentration, maternal infections, and antigen specificity. Additionally, we investigated how glycosylation patterns on antibodies affect their functionality, including both protective and pathological effects. There are several technical challenges and knowledge gaps that must be addressed in the field of the maternal–fetal antibody interface. Sample collection is complex due to ethical and practical considerations. Purifying antibodies with antigen specificity is also difficult. Because of the delicate nature of glycosidic linkages and the need for high-resolution analytical techniques, glycosylation detection necessitates sophisticated experimental equipment. Previous research has primarily concentrated on the glycosylation profiles of maternal antibodies, with few studies on maternal–fetal pairing. Finally, a thorough understanding of the intricate processes involved in placental antibody transfer and glycosylation opens new possibilities and avenues for improving maternal–fetal health, protecting neonates from infections, and mitigating the impact of autoimmune and alloimmune diseases during pregnancy.

## Figures and Tables

**Table 1:** A synopsis of IgG glycosylation profiles and their implications.

	Disease Setting	Affected-IgG	IgG-Glycan	Effector Function
Protective IgG	Healthy pregnancy	Total IgG	Increased galactosylation; Increased sialylation; Decreased fucosylation	Enhanced placental transfer
	Pertussis vaccination	Fimbriae (FIM) -specific IgG	Increased galactosylation; Increased sialylation; Decreased bisecting N-acetylglucosamine	Enhanced placental transfer; Natural killer (NK) cell activation
	Influenza vaccination	Total IgG; Hemagglutinin (HA) -specific IgG	Decreased fucosylation; Increased galactosylation; Increased sialylation;	Enhanced placental transfer; reduced inflammation
	Severe acute respiratory syndrome coronavirus 2 (SARS-CoV-2)	SARS-CoV-2-specific IgG	Increased fucosylation; Decreased galactosylation; Decreased sialylation;	Decreased placental transfer; maternal protective immunity
	Herpes simplex virus (HSV)	Anti-glycoprotein B (gB) IgG	Decreased bisecting N-acetylglucosamine; Increased sialylation; Decreased fucosylation	Decreased placental transfer; the heightening risk of neonatal disease
	Human immunodeficiency virus (HIV)	Total IgG; gp120-specific IgG	Increased fucosylation; Decreased galactosylation;	Decreased placental transfer; the heightening risk of neonatal disease
Pathological IgG	Hemolytic disease of the fetus and newborn (HDFN)	Red blood cell (RBC)-specific IgG	Increased galactosylation; Decreased fucosylation Decreased bisecting N-acetylglucosamine	Enhanced placental transfer; NK cell activation
	Fetal or neonatal alloimmune thrombocytopenia (FNAIT)	Anti-human platelet antigen (HPA)-1a IgG	Increased galactosylation; Increased sialylation; Decreased fucosylation	NK cell activation
	Systemic lupus erythematosus (SLE)	Total IgG	Decreased sialylation	The risk of fetal death
	Antiphospholipid antibody syndrome (APS)	Total IgG	Decreased galactosylation; Increased bisecting N-acetylglucosamine; Increased fucosylation	Catastrophic antiphospholipid antibody syndrome (CAPS)
